# Supplementary material for: Independent prescribing by advanced physiotherapists for patients with low back pain in primary care: A feasibility trial with an embedded qualitative component
Source: PLoS One. 2020 Mar 17;15(3):e0229792. doi: 10.1371/journal.pone.0229792 (PMC7077833; doi:10.1371/journal.pone.0229792)
Supplement: S2 File — (DOCX) [file pone.0229792.s002.docx]

Supporting Information File 3: Outcome Measures Questionnaire


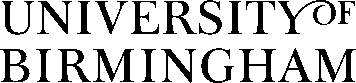


Outcome Measures Questionnaire

Participant Questionnaire

Q1 What is your gender?

- Male
- Female
- Other

Q2 What is your age?

- 17-29
- 30-39
- 40-49
- 50-59
- 60 or older


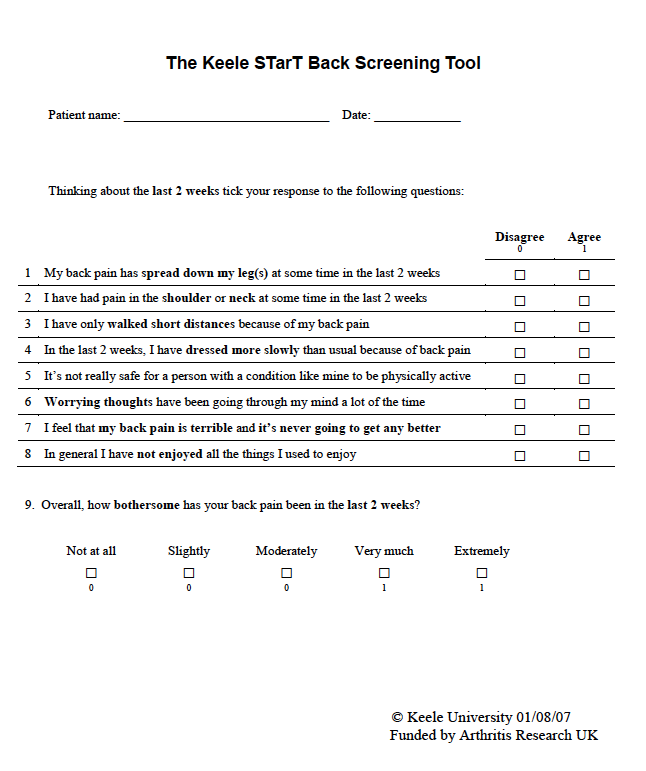


On the scales below (0-10), please mark the amount of back pain that you have experienced:

Worst pain over the last two weeks


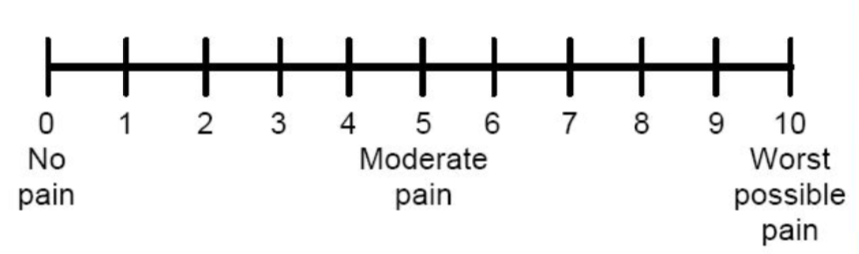


Least pain over the last two weeks


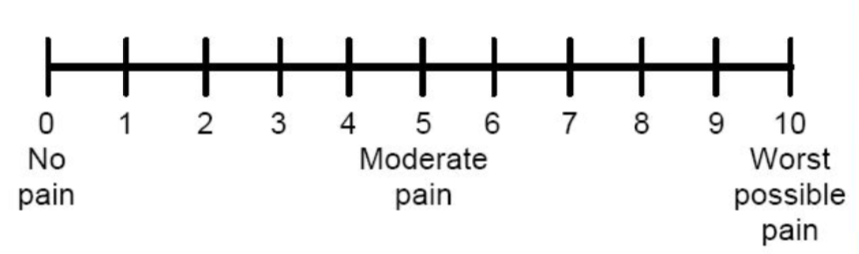


Average pain level today


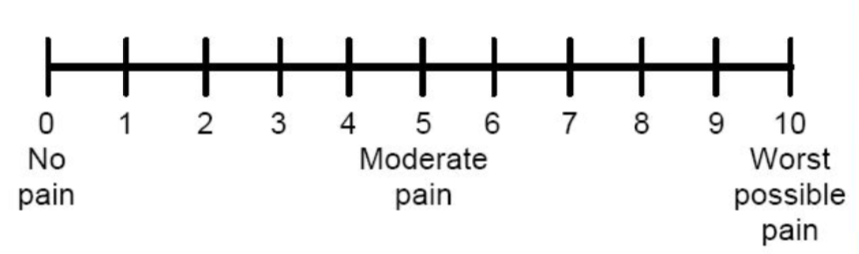


# **The Roland-Morris Disability Questionnaire**

When your back hurts, you may find it difficult to do some of the things you normally do.

This list contains sentences that people have used to describe themselves when they have back pain. When you read them, you may find that some stand out because they describe you *today*.

As you read the list, think of yourself *today*. When you read a sentence that describes you today, put a tick against it. If the sentence does not describe you, then leave the space blank and go on to the next one. Remember, only tick the sentence if you are sure it describes you today.

1. I stay at home most of the time because of my back.
2. I change position frequently to try and get my back comfortable.
3. I walk more slowly than usual because of my back.
4. Because of my back I am not doing any of the jobs that I usually do around the house.
5. Because of my back, I use a handrail to get upstairs.
6. Because of my back, I lie down to rest more often.
7. Because of my back, I have to hold on to something to get out of an easy chair.
8. Because of my back, I try to get other people to do things for me.
9. I get dressed more slowly then usual because of my back.
10. I only stand for short periods of time because of my back.
11. Because of my back, I try not to bend or kneel down.
12. I find it difficult to get out of a chair because of my back.
13. My back is painful almost all the time.
14. I find it difficult to turn over in bed because of my back.
15. My appetite is not very good because of my back pain.
16. I have trouble putting on my socks (or stockings) because of the pain in my back.
17. I only walk short distances because of my back.
18. I sleep less well because of my back.
19. Because of my back pain, I get dressed with help from someone else.
20. I sit down for most of the day because of my back.
21. I avoid heavy jobs around the house because of my back.
22. Because of my back pain, I am more irritable and bad tempered with people than usual.
23. Because of my back, I go upstairs more slowly than usual.
24. I stay in bed most of the time because of my back.

Tampa Scale:

Please mark how much you agree or disagree with the following statements:

1= Strongly disagree

2= Disagree

3= Agree

4= Strongly agree


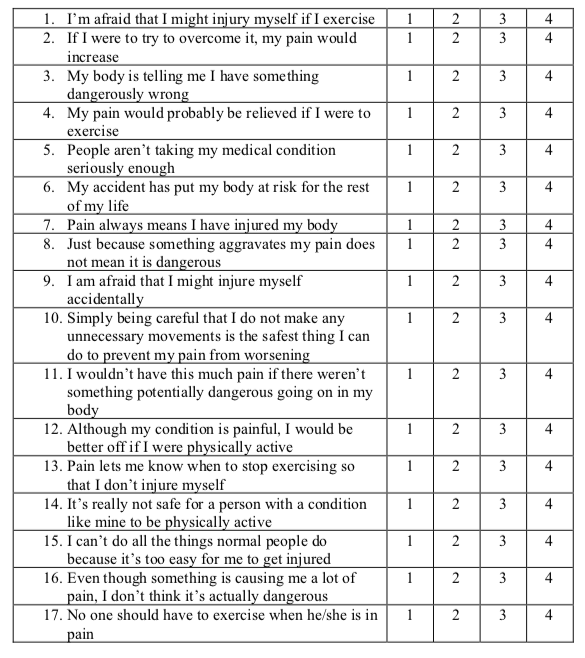


| Since receiving your prescription from the physiotherapist, how many days have you taken the medication to date? | | | | | | | |
| --- | --- | --- | --- | --- | --- | --- | --- |
| Have you seen any other healthcare professionals for your back pain since your initial assessment? | Yes | |  | | No | |  |
|  |  | |  | |  | |  |
| If YES, which type of health professional(s) have you seen, and on how many occasions? | Number of occasions | | | | | | |
|  |  | | | | | | |
|  |  | | | | | | |
|  |  | | | | | | |
|  |  | | | | | | |
|  |  | | | | | | |
|  |  | | | | | | |
| Have you had to take any time off work due to your back pain? | Yes |  | | No | |  | |
| If YES, how many days have you had to take off work due to your back pain? | | | | | | | |

Thank you for completing this questionnaire
