# Supplementary material for: Independent prescribing by advanced physiotherapists for patients with low back pain in primary care: A feasibility trial with an embedded qualitative component
Source: PLoS One. 2020 Mar 17;15(3):e0229792. doi: 10.1371/journal.pone.0229792 (PMC7077833; doi:10.1371/journal.pone.0229792)
Supplement: S3 File — (DOCX) [file pone.0229792.s003.docx]

**
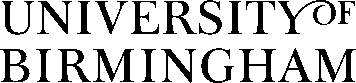
**Supporting Information File 4: Participant Information Sheet

**Participant Information Sheet: Person with Back Pain**

**Study title:** Prescribing medications for low back pain by physiotherapists

We would like to invite you to take part in a research study. Before you decide to take part, it is important for you to understand why the research is being done and what it will involve for you. The study is part of a larger PhD being completed by Tim Noblet (Researcher). Someone in our research team will go through the information sheet with you and will answer any questions that you have. Please ask if anything is not clear or if you would like more information.

**What is the purpose of the study?**

1 in 5 people with Low Back Pain (LBP) see their General Practitioner (GP) and this makes up almost 1 in 10 GP Consultations. Each year in the UK over 3 million working days are lost because almost 1 in 3 adults experience LBP at any one time. Early assessment and management of LBP is important to reduce long term problems.

The NHS is committed to providing the best services for all its patients, and due to the growing demand on health services, new and innovative ideas are being trialled to maximise quality care. A range of organisations including the British Medical Association and the Chartered Society of Physiotherapy have committed to enabling patients with LBP to be able to book appointments directly with the NHS physiotherapists in their local health centre without having to see a GP first. In addition to the normal treatment, physiotherapists are now able to prescribe medicines such as pain killers which patients usually need to get from their GP. To do this the physiotherapists complete a programme of education the same as your doctor or dentist.

Patients being able to access physiotherapists who can prescribed medicines directly is a new system in England. This study is intended to help decide how we will best assess what and what does not work, to enable provision of the best healthcare for people in England. This will be undertaken by asking approximately 30 people to complete questionnaires. A small number of people may also be asked to wear monitoring equipment (like ‘fitbits’) for a week, which assesses how active they are during each day, and 6-8 people will also be invited to participate in a focus group where they will be asked to share their opinions on how the study was conducted and how we could improve the evaluation process for the future. Physiotherapists will also have an opportunity to voice their opinions and experiences in a 1:1 interview. The results will be used to plan a large clinical trial to access how well the new services work for patients.

**Why have I been invited?**

You have been invited to take part because you have attended an appointment with the physiotherapist for your LBP and require a prescription to support your treatment. We aim to recruit 30 people across England.

**Do I have to take part?**

It is up to you to decide whether or not to take part. Feel free to ask any questions. After you have asked any questions, if you agree to take part, the researcher will ask you to sign a consent form. You are free to withdraw from the study at any time, without giving a reason. This would not affect the normal treatment that you would receive.

**What will happen to me if I take part?**

If you choose to take part in the study, you will be asked to fill out a short questionnaire on a tablet computer at your appointment with the physiotherapist. You will be asked to complete the same questionnaire 6 weeks later and 12 weeks later- these can either be sent to you by email or hard copies provided with stamped addressed envelopes so that you can return the questionnaires by post.

Some patients will also be asked to wear a small monitoring device like a ‘fitbit’ on their belt for 7 days. The monitoring devices measure the amount of time people spend moving and being still as well as your sleep pattern.

6-8 patients will be invited to attend a focus group at a local venue, and again it is up to you whether you choose to attend or not.

**What will I have to do?**

The questionnaire will take approximately 15 minutes to complete, asking you for your contact details and for information about how your back pain is affecting your everyday life at that point in time. For the 6 & 12 week questionnaire you will be able to choose either a paper (postal) or email version for you to complete. Support from your physiotherapist will always be available to you to help in completing the questionnaire.

**What are the possible disadvantages of taking part?**

It is possible that when talking about your back pain or filling in the questionnaire we may ask you to relive events which are emotional for you. However, we will make every effort to ensure that you are comfortable at all times. The only cost to you is the time needed to complete the questionnaire and (for some people) attend a focus group.

**What are the possible benefits of taking part?**

We are not able to make any promises on the benefits at this stage until we have analysed the information you provide, which may help you and other patients in the future. It will not change the treatment that you receive for your back pain.

**What will happen when the research stops?**

When the research is complete, your future treatment will not be affected in any way. Decisions about your future care will be in-line with standard procedures at the GP practice/health centre that you have been attending.

**What will happen if I don’t want to carry on with the study?**

If you do not wish to carry on with the study, you are free to withdraw at any time, without having to give a reason. Your decision to withdraw will not influence your current or future health care. It is important for us that information collected up to the point of your withdrawal is included in the analysis.

**What if there is a problem?**

It is unlikely that there will be any problems during the study. If you have a concern about any aspect of the way that you have been approached or treated during the course of this study, you can speak to Mr. Tim Noblet (researcher) or Dr Alison Rushton (Chief Investigator) who will answer any questions you have. If you remain unhappy and wish to complain formally, you can do this by following the National Health Service complaints procedure. You can get advice from the Patient Services Teams at your GP practice/ health centre (all contact details below).

In the unlikely event that you are harmed whilst participating in this study, there are no special compensation arrangements, but if this is due to someone’s negligence then you may have grounds for legal action. The normal National Health Service complaints mechanism will still be available to you. You may obtain advice from the Patient Services Teams at your GP practice/ health centre (contact details at the end of this information sheet).

**Will my taking part in the study be kept confidential?**

All information that is collected about you during the course of the study will be kept confidential. Your name or contact details will not appear on any data and you will not be identifiable from any report or publication of the findings. Your contact details will be held on a computer database so that questionnaires can be sent to you and the focus groups can be organised. This will be password protected and only accessible by the researchers. Passwords will not be used by or given to anyone outside the research team. Contact details will be destroyed at the end of the study. All information from the questionnaires that you complete and the ‘fitbits’ (if you wear one) will be kept securely by University of Birmingham for ten years following the study. After that period, all information will be disposed of in a secure manner through confidential waste.

**What will happen to the results of the study?**

Results from this study will be used to develop a clinical trial that will evaluate the use of physiotherapists who can prescribe medications in GP practices and health centres. This trial will aim to improve the patient experience and their outcomes.

The results will be published in scientific journals and through presentation at research conferences. You will not be identifiable in any report, publication or presentation. If you are interested in the results of this investigation you can obtain a summary of the results by contacting Mr. Tim Noblet or Dr Alison Rushton (contact details below).

**Who is organising and funding this study?**

The research is sponsored by the University of Birmingham and funded by Health Education England and the Private Physiotherapy Educational Fund. The research will be conducted by physiotherapists at Guys and St Thomas’ NHS Foundation Trust, the Sheffield Teaching Hospitals NHS Foundation Trust or Windemere/ Ambleside Health.

**Who has reviewed the study?**

All research in the NHS is reviewed by the Research Ethics Committee, engaged to protect your interests and those of the researchers. This study has been reviewed and given favourable opinion by the IRAS and the Research and Development Directorates, Guys and St Thomas’ NHS Foundation Trust, the Sheffield Teaching Hospitals NHS Foundation Trust and Windemere Health Centre.

**The role of the University of Birmingham**

The University of Birmingham is the sponsor for this study based in the United Kingdom. We will be using information from you in order to undertake this study and will act as the data controller for this study. This means that we are responsible for looking after your information and using it properly. The University of Birmingham securely keep identifiable information about you for 10 years after the study has finished.

Your rights to access, change or move your information are limited, as we need to manage your information in specific ways in order for the research to be reliable and accurate. If you withdraw from the study, we will keep the information about you that we have already obtained. To safeguard your rights, we will use the minimum personally-identifiable information possible.

Your physiotherapists will collect information from you for this research study in accordance with our instructions. The NHS site will keep your name and contact details confidential. If you consent to be approached to participate in a focus group, the University of Birmingham will have access to your name and contact details to arrange the focus group. The researchers who analyse the information collected will not be able to identify you and will not be able to find out your name or contact details.

The NHS site will keep identifiable information about you from this study 10 years after the study has finished.

You can find out more about how we use your information by contacting Legal Services at dataprotection@legalservices.contacts.ac.uk.

**Contact for further information or any questions about this study:**

Tim Noblet (researcher)

Tel: 07740360178

Email: TDN818@student.bham.ac.uk

Dr Alison Rushton (Chief Investigator / supervisor)

Tel: 0121 415 8597

Email: [a.b.rushton@bham.ac.uk](mailto:a.b.rushton@bham.ac.uk)

Centre of Precision Rehabilitation Spinal Pain, School of Sport, Exercise and Rehabilitation Sciences, University of Birmingham, Edgbaston, Birmingham, B15 2TT

**Site PALS Information:**

**One Medical Group-**Windermere Health Centre & Ambleside Health Centre

Telephone No:015394 45159

Email Address: [tess.shaw@onemedicalgroup.co.uk](mailto:tess.shaw@onemedicalgroup.co.uk)

**Sheffield Teaching Hospital NHS Foundation Trust**

Telephone No: 0114 271 2400

Email Address: [PST@sth.nhs.uk](mailto:PST@sth.nhs.uk)

**Guy’s and St Thomas’ NHS Foundation Trust**

Telephone No: 020 7188 8801

Email Address: [pals@gstt.nhs.uk](mailto:pals@gstt.nhs.uk)

*Thank you for taking the time to read this information sheet.*
