# Supplementary material for: Independent prescribing by advanced physiotherapists for patients with low back pain in primary care: A feasibility trial with an embedded qualitative component
Source: PLoS One. 2020 Mar 17;15(3):e0229792. doi: 10.1371/journal.pone.0229792 (PMC7077833; doi:10.1371/journal.pone.0229792)
Supplement: S4 File — (DOCX) [file pone.0229792.s004.docx]

Supporting Information File 5: Participant consent form

**
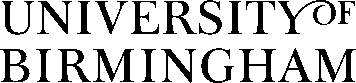
CONSENT FORM: Person with Back Pain**

**Title of Project:** Prescribing medications for low back pain by physiotherapists

Name of Participant: …………………………………………………………

**Please initial box**

1. I confirm that I have read and understand the information sheet,

for the above study. I have had the opportunity to consider the information,

to ask questions and have had these answered satisfactorily.

1. I understand that my participation is voluntary and that I am free to withdraw at

any time, without giving any reason, without my medical care or legal rights being

affected.

3. I understand that all data will be confidential and securely stored for a period of

10 years. I understand that if I withdraw from the study my data up to the point of my withdrawal will be used in the analysis

4. I agree to take part in the above study

5. I agree to be contacted to take part in the focus group

________________________ ________________ ____________________

Name of Participant Date Signature

_________________________ ________________ ____________________

Name of Person taking consent Date Signature

(if different from researcher)

_________________________ ________________ ____________________

Researcher Date Signature
