# Supplementary material for: Independent prescribing by advanced physiotherapists for patients with low back pain in primary care: A feasibility trial with an embedded qualitative component
Source: PLoS One. 2020 Mar 17;15(3):e0229792. doi: 10.1371/journal.pone.0229792 (PMC7077833; doi:10.1371/journal.pone.0229792)
Supplement: S5 File — (DOCX) [file pone.0229792.s005.docx]

Supporting Information File 6: Questions for semi-structured interviews:

| **Category of question** | **Sub-category** | **Questions** |
| --- | --- | --- |
| Intervention content and delivery | Intervention development | To what extent does the planned intervention need to be refined or adapted to make it more acceptable to APPs or more relevant or useful to LBP? |
|  | Intervention components | Consider the different aspects of the intervention e.g. completion of the outcome measures in clinic/at home, methods of completion paper/electronic, application of the accelerometer etc. Do these aspects work in the different clinical locations/different practices? If aspects of the process require changing- how can we do this to ensure interventions are delivered consistently in the full trial? |
|  | Mechanisms of action | Do you think that your advice with the additional prescription make you more confident about the outcome of your treatment?  Do you think that the presence of the accelerometer makes the patients move more? |
|  | Perceived value, benefits, harms or unintended consequences of the intervention | Did you think that being able to prescribe in your FCP clinics is useful?  What benefits/harms do you feel the addition of independent prescribing has for physiotherapists working in these roles?  Were these benefits/harms measured by the outcome questionnaire? |
|  | Acceptability of intervention in principle | Were you unhappy with any aspect of the content or delivery of the intervention? |
|  | Feasibility and acceptability of intervention in practice | What are your views about the implementation of the intervention? Has implementation varied due to the setting? Are there any important intervention-context interactions? Should implementation be tailored by setting? |
|  | Fidelity, reach and dose of intervention | Is the right amount of time etc. given to the appointments to enable the intervention to be completed to a satisfactory standard?  Were you able to adhere to the planned intervention?  If not, what are the reasons for this?  What are the limits of acceptable tailoring of the intervention? |
| Trial design, conduct and processes | Recruitment and retention | How do the planned recruitment practices work in clinical practice?  Do recruitment practices need to be improved to increase recruitment rates and levels of informed consent? If so, how?  Are clinicians willing to recruit patients, or are they uncomfortable?  Are there ways in which trial procedures could be improved to increase retention rates? |
|  | Diversity of participants | Are the planned recruitment practices likely to result in recruitment of the desired range of participants for the trial? If not, how might recruitment practices be improved? |
|  | Trial participation | How was the planned trial communication e.g. provision of the information sheet, implemented by recruiters and received by participants?  How can trial communication be improved to ensure recruiters understand patients’ views about participating in the trial? |
|  | Acceptability of the trial in principle | Is the trial design acceptable to you in principle? |
|  | Acceptability of the trial in practice | Is the trial design acceptable to you in practice, if not why? Are there ways in which you (participants/ APP) try to alter the procedures? |
|  | Ethical conduct | Are the informed consent procedures appropriate and acceptable to likely trial participants? |
|  | Adaptation of trial conduct to local context | Will the planned trial procedures allow the trial to operate effectively for patients with LBP in primary care?  Do any changes need to be made to these procedures? |
|  | Impact of trial on staff, researchers, participants and the health system | Does this trial have any unanticipated negative impacts on recruiters, participants, other stakeholders and the health system?  How can these impacts be minimised (e.g. workload involved in recruitment, numbers of measures undertaken)? |
|  | Patient and public involvement | How is patient and public involvement best achieved in the trial? |
| Outcomes | Breadth and selection of outcomes | Are outcomes important to service users selected for measurement in the full trial—both primary and secondary? Have you experienced or noticed improvements in some outcomes that need to be included in the full trial? |
| Measures | Accuracy of measures | Are the process and outcome measures valid for this participant group? |
|  | Completion of measures | Can completion rates of measures be improved? |
|  | Development of measures | Did the outcome measure used, successfully measure the variables requiring measurement?  Do we need to develop other outcome measures to be able to successfully measure the outcome of the trial? |

Questions adapted for best practice in the qualitative assessment of feasibility trials in preparation for RCTs from literature; with further development and consensus from a committee of clinicians, subject- matter and methodological experts and patients (O’Cathain et al., 2015).

O’CATHAIN, A., HODDINOTT, P., LEWIN, S., THOMAS, K. J., YOUNG, B., ADAMSON, J., JANSEN, Y. J., MILLS, N., MOORE, G. & DONOVAN, J. L. 2015. Maximising the impact of qualitative research in feasibility studies for randomised controlled trials: guidance for researchers. *Pilot and Feasibility Studies,* 1**,** 32.
