# Supplementary material for: Independent prescribing by advanced physiotherapists for patients with low back pain in primary care: A feasibility trial with an embedded qualitative component
Source: PLoS One. 2020 Mar 17;15(3):e0229792. doi: 10.1371/journal.pone.0229792 (PMC7077833; doi:10.1371/journal.pone.0229792)
Supplement: S6 File — (DOCX) [file pone.0229792.s006.docx]

Supporting Information File 7: Topic Guide for Focus Group

| **Stage of Focus Group** | **Content** |
| --- | --- |
| Introduction: Tim Noblet | PhD Candidate  Advanced Physiotherapy Practitioner  Facilitator for the Focus Group today |
| Welcome from Tim and Alison. Explanation as to the aim of the FG. | The focus group is being carried out as part of a larger feasibility trial evaluating the feasibility, suitability and acceptability of assessing the effectiveness of independent prescribing by advanced physiotherapy practitioners for patients with Low Back Pain in primary care, to inform the design of a future full trial. |
| Questions referring to Participant Information Sheet | Any questions about the project? |
| Consent | Confirm:   - All consent forms are completed - Confidentiality is fully understood by participants - All information collated is confidential - Participants will not be identified from the discussion |
| Audio Recording and transcription | Advise the participants that the FG is being audio-recorded. Recordings will be transcribed and coded. |
| Participant introduction | Introductions and setting of ground rules:   - TN to outline roles and responsibilities/ code of conduct. - Explanation re. the FG processes - The role of the observer - Agreement of ground rules – all points are valid, don’t talk over others, confidentiality |
| Questions and prompts | |

| **Objective** | **Sub-category** | **Questions** |
| --- | --- | --- |
| **General Objectives:**  Trial design, conduct and processes | Eligibility criteria | Did you feel that you were the appropriate person to take part in the trial?  If not, how might recruitment practices be improved? |
|  | Recruitment strategy | Did the recruitment process work in practice?  Do recruitment practices need to be improved to increase recruitment rates? If so, how?  Are there ways in which trial procedures could be improved to increase retention rates? |
|  | Trial participation | Were the information sheets easy to understand?  Did the information sheets provide you with enough information?  How can trial communication be improved to ensure the research team understand patients’ views about participating in the trial? |
|  | Ethical conduct | Are the consent procedures appropriate? |
|  | Adaptation of trial conduct to local context | Did the trial procedures work for the GP practice setting?  Do any changes need to be made to these procedures to make the trial run more smoothly? |

| **Objective** | **Sub-category** | **Questions** |
| --- | --- | --- |
| **Specific Objectives: Feasibility of using accelerometers** | Accelerometers | Were the accelerometers fitted easily?  Were the instructions of how and when to use the device clear? |
|  | Burden | Was wearing the accelerometer for 7 days easily achievable? |

| **Objective** | **Sub-category** | **Questions** |
| --- | --- | --- |
| **Specific Objectives: Suitability of questionnaire** | Breadth and selection of outcomes | Did the questionnaires make sense?  Were the problems that the questions asked about important to you?  Did the questionnaire miss any important points? |
|  | Accuracy of measures | Did you have enough time to complete the questionnaires?  Did you have the support you needed to be able to complete the questionnaires? |
|  | Completion of measures | How could we make it easier to complete the questionnaire? |

| **Objective** | **Sub-category** | **Questions** |
| --- | --- | --- |
| **Specific Objectives: Acceptability** | Intervention development | How does the planned trial process need to be changed or adapted to make it more acceptable to patients or more relevant or useful to people with LBP? |
|  | Intervention components | Let’s consider the different aspects of the trial e.g. completion of the outcome measures in clinic/at home, methods of completion paper/electronic, application of the accelerometer etc.  Do all these aspects work in the different clinical locations/different practices?  Do you feel different aspects of the process require changing- how can we do this to ensure interventions are delivered consistently in the full trial? |
|  | Mechanisms of action | Did the physiotherapist’s advice with the additional prescription made you more confident about the potential outcome of your treatment?  Do you think that wearing the accelerometer make you move more? |
|  | Perceived value, benefits, harms or unintended consequences of the intervention | Did you think that being able to get painkillers from your physiotherapist was valuable?  What benefits, do you feel you have experienced from the physiotherapists treatment? Were these benefits measured by the questionnaire? |
|  | Acceptability of intervention in principle | Were you unhappy with any aspect of the content or delivery of your treatment? |
|  | Acceptability of intervention in practice | Did the physiotherapists prescribing the pain killers work for you?  Could the service be provided in a better way? |
|  | Fidelity, reach and dose of intervention | Is the right amount of time etc. given to the appointments?  Were you able to adhere to the plan?  If not, what are the reasons for this? |
|  | Impact of trial on staff, researchers, participants and the health system | Does this trial have any unanticipated negative impacts on participants?  How can these impacts be minimised? |

| Prompts for further comments | Is there anything that we should have discussed that we have not?  Do you have any further views or comments that you would like to share? |
| --- | --- |
| Summary & Close | Short summary of the findings discussed in the FG  Confirm Transcription will be supplemented with field observation notes and emailed to ensure accuracy and prompt any further reflections within the next 2 weeks.  Thank participants for attending and their contributions. |

Questions adapted for best practice in the qualitative assessment of feasibility trials in preparation for RCTs from literature; with further development and consensus from a committee of clinicians, subject- matter and methodological experts and patients (O’Cathain et al., 2015).

O’CATHAIN, A., HODDINOTT, P., LEWIN, S., THOMAS, K. J., YOUNG, B., ADAMSON, J., JANSEN, Y. J., MILLS, N., MOORE, G. & DONOVAN, J. L. 2015. Maximising the impact of qualitative research in feasibility studies for randomised controlled trials: guidance for researchers. *Pilot and Feasibility Studies,* 1**,** 32.
