# Supplementary material for: Independent prescribing by advanced physiotherapists for patients with low back pain in primary care: A feasibility trial with an embedded qualitative component
Source: PLoS One. 2020 Mar 17;15(3):e0229792. doi: 10.1371/journal.pone.0229792 (PMC7077833; doi:10.1371/journal.pone.0229792)
Supplement: S7 File — (DOCX) [file pone.0229792.s007.docx]

Supporting Information File 8: Success Criteria

| **General Objectives** | **Success Criteria** |
| --- | --- |
| Eligibility criteria | A favourable number of patients fit the eligibility criteria to enable the stipulated recruitment rate |
|  | APPs agreed with the eligibility criteria |
| Recruitment strategy | Participants were recruited within the time constraints of the local clinical environment |
|  | Patients and APPs report that they were happy with the recruitment strategy |
| Data collection methods | Data were collected with ease via RedCap and no complications were experienced |
|  | Data completeness of ≥ 80 % |
|  | Patients and APPs report that they were happy with the data collection methods |
| Follow up procedures | 100% of participants were contacted for follow up |
|  | ≥80% completion of follow up outcome measures |
|  | Patients and APPs report that they were happy with follow up procedures |
| **Specific Objectives** | **Success Criteria** |
| ***Feasibility*** | |
| Participant recruitment rates | Recruitment target of n=10 per clinician met in the time available (3 months) |
| Ease of fitting accelerometers | Accelerometers were fitted within the allocated clinical time allowed with the FCP APP |
|  | Patients and APPs report that accelerometers were fitted with no issues |
| Accelerometer data collection | RedCap was able to capture the data from the accelerometers with no errors or data loss |
|  | Patients report that they were happy with data collection using accelerometers/ burden within subjectively appropriate limits |
| Capacity (time and effort) of clinicians’ complete trial related tasks | APPs report that adequate time was allowed to complete all tasks required by them during the trail |
| Training requirements required by clinicians | APPs report that they had a adequate training to be able to complete the tasks required by them during the trial |
| ***Suitability*** | |
| Outcome measures | Data completeness of ≥ 80 % |
|  | Patients and APPs report that the outcome measures were appropriate and self-explanatory |
| Compliance with wearing the accelerometers | Data collected ≥ 80 % of the requested time (16hrs/day for 7 days) |
| Time required to conduct each stage of the protocol | APPs report having adequate time to complete each stage of the protocol |
| Service infrastructure | Recruitment targets met  Data completeness of ≥ 80 % |
|  | APPs report that adequate service infrastructure is in place to allow for a full trial to be completed |
| ***Acceptability*** | |
| Intervention | Patients and APPs report that the intervention was appropriate/ satisfactory |
