# Supplementary material for: Independent prescribing by advanced physiotherapists for patients with low back pain in primary care: A feasibility trial with an embedded qualitative component
Source: PLoS One. 2020 Mar 17;15(3):e0229792. doi: 10.1371/journal.pone.0229792 (PMC7077833; doi:10.1371/journal.pone.0229792)
Supplement: Attachment — Submitted filename: Response to reviewers.docx [file pone.0229792.s010.docx]

20^th^ December 2019

Johannes Fleckenstein
Academic Editor
PLOS ONE

PONE-D-19-31324
Independent Prescribing by Advanced Physiotherapists for Patients with Low Back Pain in Primary Care: a feasibility trial with an embedded qualitative component
PLOS ONE

Dear Dr Fleckenstein,

Thank you for inviting us to submit a revised version of our manuscript addressing the points raised within the review process. Please find attached alongside this letter a copy of the revised manuscript with track changes and the clean manuscript as requested.

Please see tabulated below an outline of each change made-as raised in the reviewer comments, or our reasoning as to why a comment has not been addressed.

All authors have approved the edited manuscript and submission to PLOS One. We appreciate your time and thank you for considering this manuscript for publication. Should you have any further questions please do not hesitate to contact us.

Kind Regards,

Tim Noblet MSc, BSc (Hons) Physiotherapy, MCSP, SRP

On behalf of all authors

| Editors/Reviewers Comments | Changes made or Reason(s) for not making changes |
| --- | --- |
| Editor | |
| Please ensure that your manuscript meets PLOS ONE's style requirements, including those for file naming. | Thank you, this has been completed. |
| Thank you for submitting your clinical trial to PLOS ONE and for providing the name of the registry and the registration number. The information in the registry entry suggests that your trial was registered after patient recruitment began. PLOS ONE strongly encourages authors to register all trials before recruiting the first participant in a study. | Thank you for your comment. There has been some confusion here. The feasibility trial was registered on 11^th^ Sept 2018 (11.09.18), this date is stated on the ISRCTN website:  https://www.isrctn.com/search?q=ISRCTN15516596+  Recruitment began at the first site 28^th^ Nov 2018 after both registration (11^th^ Sept 2018) and ethical approval (30^th^ Oct 2018). |
| As per the journal’s editorial policy, please include in the Methods section of your paper: 1) your reasons for your delay in registering this study (after enrolment of participants started); 2) confirmation that all related trials are registered by stating: “The authors confirm that all ongoing and related trials for this drug/intervention are registered”. | Thank you for your comments:   1) This is not the case- please see above. 2) The required sentence has been added to page 6- Methods   The manuscript now reads:  *“To ensure transparency and reproducibility, the feasibility trial was registered on the ISRCTN database (ISRCTN15516596a-* ***registered 11^th^ September 2018****) and a detailed protocol was published (27) (S1: Published protocol).* ***The authors confirm that all ongoing and related trials for this intervention are registered.”*** |
| Please also ensure you report the date at which the ethics committee approved the study as well as the complete date range for patient recruitment and follow-up in the Methods section of your manuscript. | 1. The date that ethical approval was granted has been added- see above. 2. Date range for participant recruitment and follow-up have been added to the methods section as advised:   *Page 7: “A single-arm feasibility trial design was used to evaluate the trial objectives (26, 36). Patient reported outcome measures (S3: Outcome Measures Questionnaire) were completed digitally via an online survey at initial assessment (baseline), 6 and 12 weeks following recruitment, to enable the evaluation of the data collection tool and the feasibility of follow-up data collection* ***(Date range for participant recruitment and final follow-up: Rural town 3^rd^ December 2018-11^th^ January 2019, final follow-up 15^th^ April 2019; Regional city 28^th^ November- 19^th^ December 2018, final follow-up 13^th^ March 2019; Capital city 28^th^ February-18^th^ July 2019, final follow-up 10^th^ October 2019)*** *(34, 40).”* |
| Your ethics statement must appear in the Methods section of your manuscript. If your ethics statement is written in any section besides the Methods, please move it to the Methods section and delete it from any other section. Please also ensure that your ethics statement is included in your manuscript, as the ethics section of your online submission will not be published alongside your manuscript. | Thank you for your comment. We have edited as instructed. The Ethics statement is now included on page 6 (Methods) and reads:  ***“Ethics Approval and Consent to Participate***  *To ensure that the trial was conducted in an ethical manner within best research practice, ethical approval obtained on the 30^th^ October 2018 (IRAS project ID: 250734, Protocol number: RG_18-101, REC reference: 18/LO/1793) and HRA approval obtained, with R&D obtained from all sites (33, 34).”* |
| Reviewer 1 | |
| 1. In the Introduction the authors have presented a good rationale for the need for the trial from the current evidence base but have not given any justification for the stepped wedge trial design proposed which first appears in the Methods. A brief rationale for this trial design in the Introduction would strengthen this section. | Thank you for your comments. We appreciate your input to optimise the quality of this article.  A rationale has been added as recommended. This reads (page4-5):  ***“A stepped-wedge cluster randomised controlled trial (SWcRCT) design is proposed for use in a definitive trial owing to the contemporary nature of both the implementation of independent physiotherapy prescribing and the utilisation of APPs working as FCPs. This research design allows for the use of fewer clinicians than those required for a parallel design. It is useful in the evaluation of the implementation of new interventions, being more reflective of current practice (24-27). Although selection bias is considered a risk in cluster trials, the design is valuable when evaluating innovative clinical interventions where there is a strong ethical belief that the intervention will benefit patients.*** *A feasibility trial is required to inform the design of a definitive, low risk of bias, adequately powered, multi-centre SWcRCT investigating physiotherapist independent prescribing by APPs for patients with LBP in primary care.”* |
| 2. Similarly, within Table 1 specific objectives under Feasibility it is proposed to evaluate the ease of fitting participants with accelerometers but there is no explanation within the Introduction section to justify this. | Thank you for this comment. The following paragraph has been added to introduce the use of accelerometers.  *“The use of core outcome measures for LBP assessing pain intensity, health related quality of life and physical function are established in the literature ^60^. To date, there is no agreement regarding the ‘gold standard measures’ for each of these outcomes. Patient reported outcome measures are frequently used for assessing pain intensity, health related quality of life and some aspects of functional activity. Quality systematic reviews have revealed that the physical activity of people with LBP is lower or equal to the healthy population ^73-75^. The use of accelerometers to collect physical activity and sedentary behaviour data is advised in the literature, however the feasibility of use with patients with LBP in a trial has not been evaluated to date.”* |
| 3. There are also no objectives related to adverse events associated with prescribing and I would be interested to know if this was considered by the authors? | Thank you for this question. Adverse events were indeed considered when designing the feasibility trial. As each site followed the pre-tested and established, UK national standard governance procedures for reporting adverse prescribing events, the testing of these procedures/ methods was not deemed necessary in the feasibility trial. We agree that the collection of this data would be essential in a definitive trial design. |
| 4. Outcome measures - please clarify how participants unable to complete these measures online were dealt with and the implications of excluding people for these reasons alone | Thank you for your comments. The rationale for choosing to test the feasibility of digital data collection “*To allow real-time data capture and storage”* is reasoned in the methods. The following statements have been added to the manuscript to clarify how the data about participants that were unable to complete the surveys online were dealt with.  Methods  *“To allow real-time data capture and storage, the online outcome measures survey was built using REDCap (Research Electronic Data Capture) software hosted at the Centre for Precision Rehabilitation for Spinal Pain (CPR Spine) at the University of Birmingham, UK (53).* ***The number of participants that declined to participate as they were unable to complete the outcome measures survey online were collated to evaluate the suitability of only using digital data collection in a full trial.”***  Results  ***“No patients refused to participate owing to the inability to complete the outcomes measure survey online.”*** |
| 5. Accelerometer - Provide more details of how 10 participants were selected, and the Accelerometer data being collected and how it was fitted. Justify why there was only one data collection point as presumably in the definitive trial they will be used at follow-up and you have not tested the feasibility of this data collection method. | Details of how the 10 participants were selected has been clarified and the reason for a 7 day testing period justified. The paragraph now reads:  *To evaluate the feasibility of fitting participants with accelerometers in clinic, ease of data collection and participant compliance with wearing the accelerometer device for a 7 day period, the ten participants recruited at the rural town site had an accelerometer fitted to their left thigh for 7 days following the first consultation (33, 34). Stamped/addressed envelopes were provided to enable return of the devices after use.*  The data collected by the accelerometers has been clarified in Table 3 (Outcome measures). |
| 6. Participants - state how and who identified interested patients and the stage in their management they were identified. | Thank you for this comment. This has been clarified in the text, now reading:  *The STarT Back Tool was used* ***at initial assessment by the APPs*** *to identify patients stratified into the medium risk LBP group (5).* |
| 7. Intervention - clarify if participants had been prescribed medications by their GP prior to entry into the study. More detail of the protocol for the intervention is needed as it is not clear how it was operationalised or documented in practice regarding medication advice/prescribing/repeat prescribing. Which medications did the APP have a license to prescribe? What additional training or insurance was required to include this within their clinical practice? | We have edited the introduction to make it clear that patients do not see a GP prior to seeing a musculoskeletal FCP:  *“NHS England have mandated the introduction of musculoskeletal first contact practitioners (FCPs) in primary care. This innovation aims to enable timely access to specialist musculoskeletal practitioners such as Advanced Practice Physiotherapists (APPs),* ***without the patient first seeing a GP*** *(11, 12)*.”  To clarify the role and scope of physiotherapist independent prescribers the following text has been added to the introduction:  *“Physiotherapist independent prescribers in the UK, have completed a post-registration iNMP programme and are regulated as ‘independent prescribers’ by the Health and Care Professions Council (HCPC). They are able to prescribe, administer or direct the administration of any medication (including those unlicensed), within their individual competence, scope and expertise, for any healthcare problem.”*  Collecting data regarding ‘what’ the APPs prescribed was not within the scope of the feasibility trial which aimed to evaluate the methods specified in the objectives. |
| 8. Outcomes - clarify the restriction in technology that prevented sleep measurement. | Wording has been altered to clarify the validity limitations associated with assessing sleep with accelerometers.  *“Assessment of sleep via accelerometer was detailed in the published protocol (36), unfortunately, limitations in validated accelerometer technology measuring sleep available at the time of testing prevented evaluation. This was the only deviation from the feasibility trial protocol.”* |
| 9. Sample size -the target recruitment rate of 10 participants per APP over a 6 month period seems very low and unrealistic for testing feasibility for a definitive trial. Please provide justification for this. | Thank you for this comment. This is justified by the literature on page 12.  *“A sample size of n>20 is regarded as adequate within the literature, when testing feasibility objectives for cRCTs, however a total sample of n=30 participants was planned to allow for under-recruitment within the specified time period and loss to follow up (33, 34, 44, 45).”* |
| 10. Qualitative Studies APP interviews - could the authors provide the interview schedule for these interviews for consistency with the Focus Group topic guide which is provided | The topic guide for the interviews has now been included. |
| 11. Integration - within the methods reference is made to the a priori success criteria -but these are not evident until further into the Results section - reference to Table 10 and/or a short synopsis in the text would be useful at this point in the manuscript. | The success criteria have been submitted as a supplementary file due to the number of tables within the manuscript. To clarify, the following text has been added:  *“Following quantitative and qualitative analysis, data were assessed against a priori defined success criterion developed by experts and informed by the methodological literature (43, 58, 89).* ***Success criteria can be found in supplementary file 8 (S8: Success Criteria).*** *Trial objectives were considered successful if the success criteria were satisfied following the integration of the quantitative and qualitative findings (34, 89).”* |
| 12. The recruitment rate of two practices appears notably higher than the target 6 months - could you clarify this in the text | This has been actioned. Text now reads:  *“Two sites recruited the pre-defined n=10 participants within the 6 month recruitment period (3 and 4.5 weeks). The capital city site recruited n=9 participants over the 6 month period.”* |
| 13. Flow chart - please check the n= under Fulfilled eligibility criteria - as it states 7 participants were excluded but 29 were allocated to the Intervention - should this be 36? | Thank you for spotting this error. The flow diagram has been updated to read n=36. |
| 14. In reporting loss to follow-up rates please state the expected rates were 20% a priori in the text | Thank you for this comment. This has been actioned in the text.  *“Successful loss to follow up was defined a priori as <20%. 48% of participants were lost to follow up at 6 weeks, with 65.5% at 12 weeks (Fig 1). One site had a loss to follow up of 89%, suggestive of site-specific issues.”* |
| 15. Accelerometry Add the gender of n=10 participants  Clarify if there were any missing data or non-wear times | Actioned in the text as per comments:  *“Ten participants* ***(n=2 male, n=8 female****) wore an ActivPal accelerometer 24 hours a day for seven days. Data collected by the accelerometers are displayed in Table 6.* ***There were no missing data.*** *Participants spent an average of 18.57hrs (SD=1.54) sitting per day, 4.14hrs (SD=1.17) standing and 1.3hrs (SD=0.39) walking. Participants completed 5884.66 steps per day (SD=2255.11), with a mean activity score of 32. 94MET.h (SD=1.03).”* |
| 16. Please provide some demographic details of the three APPs - age, level of experience, current job title and postgraduate qualifications | This has been completed as requested. |
| 17. Recruitment - could the authors discuss how selection bias was minimised in this feasibility trial as APPs were recruiting | Thank you for your comment. The following text was added to the methods to clarify (page 9-10):  *“Convenience sampling was used as this method has the advantages of fluid recruitment. To minimise selection bias, patients fitting the eligibility criteria were recruited consecutively (101).”* |
| 18. Follow-up - As all participants returned Accelerometers in person the trial has not demonstrated the feasibility of using postage as a follow-up method which should be acknowledged in the text. | Thank you for this comment. We have added the following text as requested:  *“All participant returned the accelerometer in person, therefore the feasibility of returning the device by post was not formally evaluated.”* |
| 19. Qualitative Interviews - Patients Clarify if the n=6 participants were compliers or drop-outs and if compliers, how reasons for drop-out will be identified and dealt with in the definitive trial. | To clarify compliers and drop-outs the following was added to the text:  *“Six participants from the trial component of the feasibility trial consented to participate in the focus group. Purposive sampling enabled a representative spread of ages. 66% (n=4) of the participants were female, 66% (n=4) of participants completed the feasibility trial, with 33% (n=2) lost to follow up at 12 weeks.”* |
| 20. Eligibility criteria - Discuss the implications of including all three STarT back subgroups in a definitive trial as proposed.  State the proposed long-term follow-up time point(s) being considered for a definitive trial. | Thank you for your comments. The implications of including all STarT back groups are spread throughout the different sections of the discussion to illustrate points e.g. the effects on recruitment rates:  *“However, it is posited that with the expansion of the proposed eligibility criteria to include all patients with non-specific LBP +/- leg pain, the full scope of physiotherapist prescribing and the adoption of additional recruitment capacity via research assistants and administrative staff, that recruitment rates and retention at all sites would be acceptable.”*  As specified within the design and discussion sections, this feasibility trial did not attempt to estimate proposed sample size. The rationale for this is explained within the Methods- design section:  *“Two-arm feasibility studies aiming to calculate intra-cluster correlation coefficients (ICCs) required for sample size calculations have been shown to exhibit insufficient accuracy (44). Therefore, a prospective, mixed-methods, single-arm feasibility trial, exclusive of sample size estimation was employed to evaluate the trial objectives on the experimental arm of the future SWcRCT (33, 43, 45).”*  Additional words have been added regarding rescheduling the proposed primary end point for a definitive trial:  *“If a definitive trial is to include all STarT Back stratification groups, additional longitudinal follow up procedures should be incorporated, rescheduling the trial primary endpoint to 1 year to allow for evaluation of patients in the long term.”* |
| 21. Explain how the prescribing practice of APPs was recorded in this feasibility trial and would be recorded in a definitive trial as this is not evident from the data presented and is a key variable to report to demonstrate APPs prescribing practice. | The evaluation of prescribing practice was therefore not in the scope of this feasibility trial, but is being addressed in a parallel audit. |
| 22. It would also be informative to include literature on the views of GPs on APPs taking on a prescribing role and how it is proposed this role will be shared in future practice. | We agree, but unfortunately, this literature does not exist to date. |
| 23. The statement that a definitive trial is feasible with minor modifications needs some tempering as there are alot of proposed changes to the trial protocol including increasing its eligibility criteria, increasing the number of outcome measures, follow-up methods and follow-up points and providing additional training of APPs and recruiting and training research assistants to name a few, some of which will need to be piloted. I would consider these to be more than minor modifications and would encourage the authors to revise this wording in the Discussion, Conclusions and Abstract. | The ‘minor’ refers to the size of the changes required rather than the number of changes required. This fits with terminology used by the NIHR in the UK. Following discussions with the trial steering committee including PPI representation, authors would like to maintain the wording of ‘minor’. |
| Reviewer 2 | |
| To my opinion, this is an excellent manuscript about an interesting study. However, I recommend the authors to look closer on following:   1. ensure that all abbreviations are explained the first time they occur. | Thank you for your comments. We appreciate your input to optimise the quality of this article.  Thank you for your comment, this has been actioned. |
| 2. help the readers to interpret the primary and secondary outcomes measure data, both in the text and in the Table 5. | Additional words have been added based on this recommendation. The text now reads:  *“Table 5 presents mean primary and secondary outcome measure data collected from the outcome measure questionnaire with variability reported by the use of standard deviations (SD). Reductions in pain were found for all pain categories as time progressed. Mean scores on the RMDQ reduced from 9.21 (SD 5.58) at base line to 8.07 (SD 5.82) at six weeks, then increased to 9.70 (SD 5.33) at 12 weeks. Between baseline and 12week, improvements were seen across all components of the secondary outcome measures other than anxiety and depression (EQ-5D 5L) which increased with time. No participants scored the distinct lower limit in any of the outcome measures; therefore, no floor effects were found. As primary and secondary outcomes improved, absence from work and prescription utilisation reduced.”* |
| 3. demographic data of the participating APP seem to be missing. | This has been added as requested. |
| Reviewer 3 | |
| 1. The authors seem to have tried to combine a desire to pilot their future trial and assess the feasibility of the intervention... however, their design only permits them to assess the feasibility of their intervention... I can understand that the stepped wedge component may have been difficult to do but I was unclear why a cluster feasibility pilot trial design was not undertaken? If the objective was not to pilot the future trial but to only examine the feasibility of intervention - then its unclear why the authors report success criteria for the recruitment and retention rates? | Thank you for your comments. We appreciate your input to optimise the quality of this article. In planning this trial we have carefully considered the issue you raise.  The NIHR (UK) state that “Feasibility studies are pieces of research done before a main study in order to answer the question ‘Can this study be done?’” and states that a feasibility trial often consists of evaluating 1 arm of the planned definitive trial. As specified in the article, the study was designed as a feasibility trial to evaluate specific design objectives aiming to answer the question- can this study be done? Further rationale for this has been added to the introduction as per reviewer 1. The success criteria were reported for the recruitment and retention rates to aid in the evaluation of the general objectives assessing the feasibility, suitability and acceptability of the recruitment and follow up strategies, not the evaluation of the ‘feasibility of the intervention’ (physiotherapist independent prescribing). |
| 2. The authors have chosen an RCT design to test an intervention of something that is not a new or novel intervention, but actually a different professional role thats part of a new service delivery approach - thats very specific to the NHS in the UK. I was therefore uncertain about the wisdom of a trial design and of the international relevance of the study. I wonder if a service evaluation might have been a better design? | Thank you for your comment. The 4 expert peer reviewers that reviewed the feasibility trial protocol and reviewers 1 & 2 have commented on the merits of evaluating the novel use of physiotherapist independent prescribing for LBP in primary care by APPs.  To further clarify the rationale for evaluation by trial, additional reasoning has been added to the background section- see response to reviewer 1. The rigor of a definitive trial is required to ensure low risk of bias findings regarding effectiveness.  Although physiotherapist independent prescribing is only legal in the UK at present, other countries such as Australia, Nigeria and South Africa are considering the introduction. The World Confederation of Physical Therapy (WCPT), part of the World Health Organisation (WHO), are closely involved in evaluating the potential benefits of physiotherapist prescribing internationally. https://www.wcpt.org/policy/ps-advanced-pt-practice |
| 3. If the authors feel this is a new intervention worth testing for effectiveness, then some background information on how it was developed and the key targets of the intervention. | Thank you for your comments. These comments are reflective of reviewer 1. The authors have added detail to the ‘Background’ section of the article for clarification. Please see responses to reviewer 1. |
| 4. It was unclear how and where patients were recruited and identified and from what denominator of those invited? | Thank you for your comment. Please see response to reviewer 1, comment number 6. |
| 5. A major concern for cluster trials is recruitment selection bias. This was not mentioned. | Thank you for your comment. Please see response to reviewer 1, comment number 17. |
| 6. What was the purpose of collecting accelerometer data in relation to the feasibility study? Was that a key treatment target? Or was that actually piloting an outcome for the main trial? | The purpose of collecting the accelerometer data was to “*evaluate the ease of fitting participants with accelerometers and ease of data collection”*, as per the specific objectives. If the use of accelerometers is to be feasible it was essential to ensure that no data was lost. To clarify this finding additional text has been added in the results section:  *“Ten participants* ***(n=2 male, n=8 female****) wore an ActivPal accelerometer 24 hours a day for seven days. Data collected by the accelerometers are displayed in Table 6.* ***There were no missing data.*** *Participants spent an average of 18.57hrs (SD=1.54) sitting per day, 4.14hrs (SD=1.17) standing and 1.3hrs (SD=0.39) walking. Participants completed 5884.66 steps per day (SD=2255.11), with a mean activity score of 32. 94MET.h (SD=1.03).”* |
| 7. The authors collected a range of outcome measures they plan to use within a future main trial – but these seemed to be primarily to be about piloting them for the main trial and were largely unrelated to questions about the feasibility of the intervention. | Thank you for your comment.  Collecting the range of outcome measures enabled the feasibility trial to assess:   - the feasibility, suitability and acceptability of the proposed full trial (32) including the data collection methods. - the capacity (time and effort) of clinicians and researchers to complete trial related tasks - the time required to conduct each stage of the protocol   These are explicit in the feasibility trial objectives and are essential to evaluate “whether the trial can be done” as per the NIHR (UK) definition of feasibility studies/trials. |
| 8. The qualitative aspects seem to lack any theoretical framework… | The qualitative aspects follow a grounded theory- thematic analytical framework as detailed in the methods section:  *“A grounded theory theoretical framework enabled a thematic analytical approach to analyse and synthesise the qualitative data. This method enables identification of the important thoughts and views of the population being studied, providing explanations alluding to how the concerns may be resolved or processed in preparation for a full trial (43, 85, 86). Transcripts were coded line-by-line using NVivo 11 software (QSR International, Melbourne, Australia) by one researcher (TN) and verified by a second researcher (AR) (49, 86, 87). Rigorous comparative analysis was completed to identify similarities and differences within the data, informing the development of descriptive categories which were linked, merged or split to synthesise a conceptual understanding of the data (86, 87). To avoid single researcher bias, the second researcher (AR) re-interrogated the data to validate or contradict findings. Following this process, to ensure trustworthiness, outcomes were discussed with a panel of experts for confirmation and agreement (85, 86, 88).”* |
| 9. In the results it appears that physical function decreased over 3 months and that anxiety and depression worsened…. How do these disappointing results suggest feasibility or any promise from the intervention? | As stated in the article, the feasibility trial was not powered to evaluate efficacy. Researchers are therefore unable to conclude any cause or effect based on the data collected. The positive findings within the qualitative data promote the need for a definitive trial to evaluate effectiveness. The trial should integrate the findings from this feasibility trial and the pilot studies recommended to ensure quality and low risk of bias. |
| 10. The attrition rates at 12 weeks were awful…. unacceptably low. | The authors agree with this statement and have discussed this in detail within the article, alongside potential changes to the design that aim to improve dropout. It is recommended that these changes are piloted prior to a full trial (discussion/conclusion). |
| 11. Table 5, I wondered why the EQ-5D utility score was not given? | The feasibility trial was not powered to assess health utility therefore the EQ-5D-5L UI was not processed. |
| 12. For an intervention study about prescribing - the measure called "Total prescription utilisation" seems a very blunt and inadequate measure… Could we not know what APPs prescribed for these patients? Perhaps provided in categories such as NSAIDs, opioids, atypical analgesics etc… | A parallel multi-site service evaluation is on-going at present collecting this data. The authors acknowledge that in a definitive trial prescribing data will be key. It is acknowledged that this data would be collected in a definitive trial but was not an objective needing evaluation in this feasibility trial. This information should be collected in the piloting of the data collection tool recommended within the discussion and conclusion of the article. |
