# Supplementary material for: Independent prescribing by advanced physiotherapists for patients with low back pain in primary care: A feasibility trial with an embedded qualitative component
Source: PLoS One. 2020 Mar 17;15(3):e0229792. doi: 10.1371/journal.pone.0229792 (PMC7077833; doi:10.1371/journal.pone.0229792)
Supplement: Attachment — Submitted filename: Response to reviewers.docx [file pone.0229792.s011.docx]

4^th^ February 2020

Johannes Fleckenstein
Academic Editor
PLOS ONE

PONE-D-19-31324
Independent Prescribing by Advanced Physiotherapists for Patients with Low Back Pain in Primary Care: a feasibility trial with an embedded qualitative component
PLOS ONE

Dear Dr Fleckenstein,

Thank you for inviting us to submit a revised version of our manuscript addressing the points raised within the review process. Please find attached alongside this letter a copy of the revised manuscript with track changes and the clean manuscript as requested.

Please see tabulated below an outline of each change made-as raised in the reviewer comments, or our reasoning as to why it has not been possible to address a comment.

All authors have approved the edited manuscript and submission to PLOS One. We appreciate your time and thank you for considering this manuscript for publication. Should you have any further questions please do not hesitate to contact us.

Kind Regards,

Tim Noblet MSc, BSc (Hons) Physiotherapy, MCSP, SRP

On behalf of all authors

| Editors/Reviewers Comments | Changes made or Reason(s) for not making changes |
| --- | --- |
| Reviewer 1 | |
| The authors have addressed the majority of my comments comprehensively. There are a few minor changes related to three responses that would increase the accuracy of the manuscript as detailed below: | Thank you for your feedback to optimise this article. |
| Point 2 - the last sentence in the response is not an accurate reflection of the literature as the feasibility of using ActivPal accelerometers with LBP patients has been previously tested in the following trial. McDonough SM, Tully MA, Boyd A, O'Connor SR, Kerr DP, O'Neill SM, Delitto A, Bradbury I, Tudor-Locke C, Baxter GD, Hurley DA. Pedometer-driven walking for chronic low back pain: a feasibility randomized controlled trial. Clin J Pain. 2013 Nov;29(11):972-81. doi: 10.1097/AJP.0b013e31827f9d81. | Thank you for your comment. For clarity the manuscript now reads (page 5):  *“The use of accelerometers to collect physical activity and sedentary behaviour data is advised in the literature, however although the feasibility of use with patients with chronic LBP has been established (32), the feasibility of fitting accelerometers for LBP in the context of an FCP clinic has not been evaluated to date.”* |
| Point 8 - The response concerning the limitations in validated accelerometer technology does not explain the issue that prevented the recording of sleep data. Further rewording is recommended. | Wording has been altered to clarify (page 11):  *“Assessment of the quality of sleep via accelerometer data was detailed in the published protocol (37). Unfortunately, the devices available for testing the feasibility of fitting the accelerometers in FCP clinics and evaluating participant’s tolerance of wearing the devises, were not validated for measuring quality of sleep. Previous quality research has established that it is feasible to evaluate time sleeping using the data collected by the devises when cross referenced with a participant diary mapping activity, sedentary time and time asleep. It was not deemed necessary to re-evaluate this process (32, 65). This was the only deviation from the feasibility trial protocol.”* |
| Point 15. The reporting of mean recorded data for sitting, standing and walking equates to 24hrs and does not take account of sleep time. Linked to the previous response there is a need to state how sleep can be differentiated from awake time for other researchers using this device. | Thank you for your comment. To clarify this point to the reader the following words have been added within the secondary outcomes section to complement the wording added to page 11 (page 12):  *“An accelerometer will be used to collect data including: time sitting, standing and walking, steps count and overall activity score (78).* ***Where necessary, participant diaries mapping activity, sedentary time and time asleep can be used to differentiate between sedentary time and sleep (78).*** *To date no individual brand/model of accelerometer has been identified as gold standard.”* |
